# *In vitro* inhibition of hepatic stellate cell activation by the autophagy-related lipid droplet protein ATG2A

**DOI:** 10.1038/s41598-018-27686-6

**Published:** 2018-06-18

**Authors:** Yun Hong, Sirui Li, Jifeng Wang, Youming Li

**Affiliations:** 1 First Affiliated Hospital, School of Medicine, Zhejiang University, Hangzhou, China; 2 Institute of Biophysics, Chinese Academy of Sciences, Beijing, China; 3 University of Chinese Academy of Sciences, Beijing, China

## Abstract

Clinical studies have found that moderate intake of retinol or oleic acid can enlarge the lipid droplets of hepatic stellate cells and suppress their activation. However, the link between lipid droplets and cell activation is unknown. This study compared the dynamics of lipid droplet-associated protein expression between activated and reverted stellate cells. Reversion of the activated human stellate cell line LX-2 and inhibition of primary mouse stellate cell activation were induced by retinol or oleic acid, which resulted in larger lipid droplets and the downregulation of cell activation markers. Quantitative proteomics and immunoblotting were performed to compare lipid-droplet protein profiles between activated and reverted LX-2 cells. Compared to expression in activated cells, 50 lipid-droplet proteins were upregulated, whereas 28 were downregulated upon reversion. ATG2A was significantly enriched in lipid droplets of retinol/oleic acid-treated LX-2 cells and quiescent primary stellate cells. Reduced expression of α-SMA, increased expression of perilipin-3, enlarged lipid droplets, and suppression of autophagic flux were observed in ATG2A-deficient LX2 cells. Lipid-droplet protein profile changes during the reversion of activated stellate cells might provide new insights into the molecular mechanisms linking lipid droplets to liver fibrosis. ATG2A could represent a potential new drug target for hepatic fibrosis.

## Introduction

Liver fibrosis, a major world health problem, is characterized by the excessive deposition of extracellular matrix (ECM), which distorts hepatic architecture and finally resulted in cirrhosis and liver failure^1–4^. Current evidence demonstrates that the main producers of fibrotic ECM are hepatic stellate cells (HSCs), which transform into a myofibroblast-like phenotype during liver fibrosis^5,6^. The loss of large amounts of lipid droplets (LDs) containing retinol ester (RE) and triacylglycerol (TAG) is a hallmark of HSC activation^7,8^. Several experimental and clinical studies have revealed that moderate dietary intake of retinol or oleic acid (OA) can inhibit hepatic fibrosis^9–14^. *In vitro* studies have also demonstrated that retinol and OA are able to induce lipid accumulation and LD enlargement in HSCs^15–17^. In addition, the expression of GFAP, which partially indicates a quiescent state in stellate cells, is maintained by retinol^16^; further, the expression of α-SMA (alpha smooth muscle actin), a sensitive and reliable marker of activation, is suppressed by OA^14^. These results suggest a potential role for lipid metabolism in the regulation of HSC activation, or vice versa, as demonstrated by recent *in silico* analyses^18,19^.

The LD, which serves as a neutral lipid reservoir and the center of lipid metabolism, is a ubiquitous organelle. Proteins regulating the dynamics of LDs also play roles in most prevailing diseases such as NAFLD^20^, myocardial dysfunction^21^ and insulin resistance in skeletal muscle^22^. Recently, Mahony *et al*.^23^ expounded that Rab18 is a retinoid-responsive LD protein and its mRNA, protein and LD colocalization increased during HSC activation. Knockdown of Rab18, deletion of its GTPase activity, or prevention of its insertion into LDs can retard the loss of LDs and block the activation of HSCs. Upregulation of ADRP (perilipin-2/Plin2), which coats LDs and regulates their formation and lipolysis, was shown to correlate with the downregulation of HSC activation in the LX2 cell line^17^. Additionally, exogenous perilipin-5 (Plin5) expression in primary HSCs was shown to reverse the activation phenotype and promote LD formation^24^. Frequently, several hundred different proteins can be detected in LDs and their biological roles have become significantly broader than previously anticipated (e.g. DNA replication, transcription and translation, membrane trafficking, and cell signaling)^25^. Recently, expanding roles for LDs, specifically as signaling vehicles across nuclear, inter-, and intracellular transporters of fatty acids and as an arena between proteins and intracellular pathogens have been reviewed^26,27^. Considering the large numbers of proteins and broad roles of LDs, we hypothesized that LDs might be involved in the complex regulation of HSC activation beyond these mechanisms described previously^17,23,28^.

In the present study, retinol and OA were found to promote the reversion of activated HSCs and retard the spontaneous activation of primary mouse HSCs (mHSCs) by inducing changes in LDs. Tandem mass tag (TMT) labeling plus liquid chromatography–mass spectrometry (LC-MS/MS), a cutting edge quantitative proteomic technique, was further applied to extensively analyze the proteomes of LDs purified from LX-2 cells reverted by retinol or OA. Among 78 differentially expressed LD proteins, ATG2A, a protein known to be associated with autophagy, was selected to explore its role in activation through siRNA-mediated knockdown.

## Materials and Methods

### Materials

The key reagents used in this study are listed in Table S1.

### *In vitro* assays

The LX-2 human stellate cell line and primary mouse HSCs were used for *in vitro* assays. Primary mHSCs were isolated from 24-week-old male C57BL/6 mice by *in situ* collagenase-pronase perfusion and subsequent centrifugation using OptiPrep (SIGMA) density gradients (8.2–17.5%)^29^. The purity of isolated mHSCs was assessed by fluorescent microscopy using autofluorescence and LipidTOX Red (Thermo Scientific, Waltham, MA) staining^29,30^ (Fig. S1A). LX-2 cells were cultivated in DMEM (Macgene Biotech, Beijing) with 10% FBS and 100 U/ml of penicillin and streptomycin at 37 °C in an atmosphere of 5% CO_2_. Upon reaching 50–60% confluence, cells were starved in serum-free DMEM for 24 hours. Cells were stimulated with 10 μM retinol (Sigma) or 100 μM OA (Sigma) in DMEM containing 2% FBS for 24 hours. Preparation of OA solution was performed according to Liu *et al*.^31^. Primary mHSCs were treated with retinol, OA, or vehicle after attaching (approximately 2 hours of incubation). The morphology of cells was observed by confocal microscopy (FV1000). Areas of cells and LDs were measured using ImageJ software 1.48 V^32^. Briefly, images were converted into binary images in which LDs and cells were represented only by pixels. The area of LDs was calculated using the default setting, whereas cell areas were calculated based on manually inputted outlines due to their irregular shapes.

### LD isolation and analysis

Isolation of LDs from LX-2 cells was performed according to Liu *et al*.^33^. Briefly, cells were lysed in buffer A containing 25 mM tricine and 250 mM sucrose (pH 7.6), followed by N_2_ cavitation (500 psi for 15 minutes on ice). All the homogenate were centrifuged at 1000 × g to remove the debris and nucleus. The postnuclear supernatant (PNS) fraction overlaid with 2 mL of Buffer B (20 mM Hepes pH 7.4, 100 mM KCl, 2 mM MgCl_2_) was subjected to centrifugation at 20,000 × *g* for 1 hour at 4 °C, and the white band containing LDs at the top of gradient was collected. Further purification was performed by centrifuging (20,000 × *g*, 3 minutes) the LD resuspension in buffer B. The precipitation after ultracentrifugation was collected as total membrane (TM). The cytosol fraction was obtained from the supernate above the TM. LD protein profiles from three independent LD isolations for each treatment were assessed by silver staining gels, and some distinct differences among the three treatments could be observed. LD purity was validated by silver staining, immunoblot assays, and mass spectra analyses according to previous research^21,33^. The sizes of isolated LDs were measured using a Delsa Nano C particle analyzer^33^. Lipid composition was analyzed by thin layer chromatography (TLC)^34^.

### Immunoblot assay and reverse transcription quantitative real-time PCR (RT-qPCR)

Protein samples for SDS-PAGE gel electrophoresis were prepared as follows. LD proteins were precipitated using a mixture of 70% acetone and 30% chloroform. The precipitated LD proteins, as well as other subcellular fractions, were dissolved in 2× SDS sample buffer (125 Mm tris base, 20% glycerol, 4% SDS, 4% β-mercaptoethanol, and 0.04% bromophenol blue). Whole cells were lysed directly in 2× SDS sample buffer followed by three rounds of sonication at 200 W for 3 seconds. Samples were denatured at 95 °C for 5 minutes, which was followed by centrifugation at 10,000 × *g* for 30 seconds. Ten microliters of proteins were separated by SDS-polyacrylamide gel electrophoresis and electro-transferred to PVDF membranes, which were blocked with 5% non-fat milk and then probed with primary antibodies. Visualization of protein bands was performed with enhanced chemiluminescence substrate (PerkinElmer) after probing with secondary antibodies. GADPH was used as an internal control. Image analysis was performed with ImageJ. Total RNA was isolated from LX-2 cells using TRIzol reagent (Invitrogen). RNA was quantified using NanoDrop 2000 (Thermo Fisher Scientific). 2 µg of total RNA were digested by RNase-Free DNase (Promega, M610A) and then reversed transcripted into cDNA by M-MLV Reverse transcriptase (Promega, M170B) according to the manufacturer’s protocol. The RT-PCR was performed in duplicates using TransStart Top Green qPCR SuperMix (Transgen BioTech, AQ131). The polymerase was activated for 30 seconds at 94 °C then PCR cycling for 42 cycles of 15 seconds at 94 °C, 15 seconds at 60 °C and 25 seconds at 72 °C with a final extension at 72 °C for 10 minutes. The melt curve was performed from 60 °C to 95 °C. Primers were synthesized by Sangon Biotech Co., Ltd. The PCR primer sequences and Tm values are provided in Table S2. GAPDH was used as internal control for mRNA assays. The fold change of genes mRNA expression was calculated by the equation 2^−ΔΔCt^ ^35^.

### Quantitative proteomics of LD proteins and bioinformatics analysis

Quantitative proteomic analysis was carried out using a previously reported method^36^ and an overview of workflow was provided. In short, LD proteins precipitated by acetone and chloroform were digested with trypsin (50:1) at 37 °C overnight. Then the peptides was desalted on OASIS HLB column and eluted with 60% acetonitrile. The lyophilized peptides via vacuum centrifugation were labeled by six-plex TMT TAILS (terminal amine isotopic labeling of substrates) according to the manufacturer’s protocol (Thermo Scientific). Equal amounts of peptides from each biological replicate of different experimental conditions were labeled as follows: TMT-126/−127 was used for control samples, TMT-128/−129 for Retinol-treated samples and TMT-130/-131 for OA-treated samples. Q Exactive mass spectrometer (Thermo Scientific) equipped with an Easy-nLC 1000 HPLC system (Thermo Scientific) was utilized in this nanoLC-MS/MS experiment. The labeled peptides were loaded onto a 100 μm id × 2 cm fused silica trap column packed in-house with reversed phase silica (Reprosil-Pur C18 AQ, 5 μm, Dr. Maisch GmbH) and then separated on an a 75 μm id × 20 cm C18 column packed with reversed phase silica (Reprosil-Pur C18 AQ, 3 μm, Dr. Maisch GmbH). The raw data from Q Exactive were analyzed with Proteome Discovery version 1.4.1.14 using Sequest HT search engine for protein identification against the Uniprot human protein database (updated on 02–2016). FDR analysis was performed with Percolator and FDR <1% was set for protein identification^37^. The peptides confidence was set as high for peptides filter.

Among the 496 total LD proteins, differentially regulated proteins (P < 0.05) were identified by multiple ANOVA analysis in conjunction with Dunnet’s analysis using R (3.2) and their associations with pathways of HSC activation/deactivation were explored using KEGG (www.kegg.jp). Briefly, genes in each pathway for *Homo sapiens* were extracted from the KEGG database. Detected protein sequences corresponded to these genes using a standalone Tblastn analysis. Fish’s extract test was further applied to identify enriched pathway (unadjusted p < 0.05). Differentially expressed LD proteins were further validated by immunoblotting.

### *siRNA* silencing of ATG2A in LX-2 cells and primary mHSCs

LX-2 cells, grown to 50% confluence in 10% FBS medium, were transfected with human ATG2A siRNA (GenePharma, China) using Lipofectamine RNAiMAX (Invitrogen, USA) following the instruction of the manufacturer. Primary HSCs cultivated for 24 hours after isolation were transfected with mouse ATG2A siRNA (GenePharma, China) and transfection was repeated 3 days later, which was followed by harvesting at day 7. Transfection with medium only and scrambled siRNA were used as negative controls.

### Statistical analysis

Significant differences in lipid composition, LD quantification and protein expression among treatments were compared by ANOVA analysis in conjunction with Dunnet’s analysis using R (3.2). Data were presented as mean ± SD (standard deviation calculated from independent samples). Different letters (a, b, c) over the bars indicate significant differences between treatments (unadjusted p < 0.05).

### Data availability

All data generated or analyzed during this study are available upon request. All data generated or analyzed during this study are included in this published article (and its supplementary information files).

### Ethics approval and consent to participate

All experimental procedures involving mice were approved by the Animal Care and Use Committee of the Institute of Biophysics, Chinese Academy of Sciences, Beijing, China, which has permission to conduct animal experiments, SYXK (SPF 2009–111). All experimental protocols conformed to the Guide for the Care and Use of Laboratory Animals (NIH Publication Eighth Edition, updated 2011).

## Results

### Retinol/OA-induced enlargement of LDs is accompanied by the reversion of activated human stellate cell line LX-2

**Figure 1:**
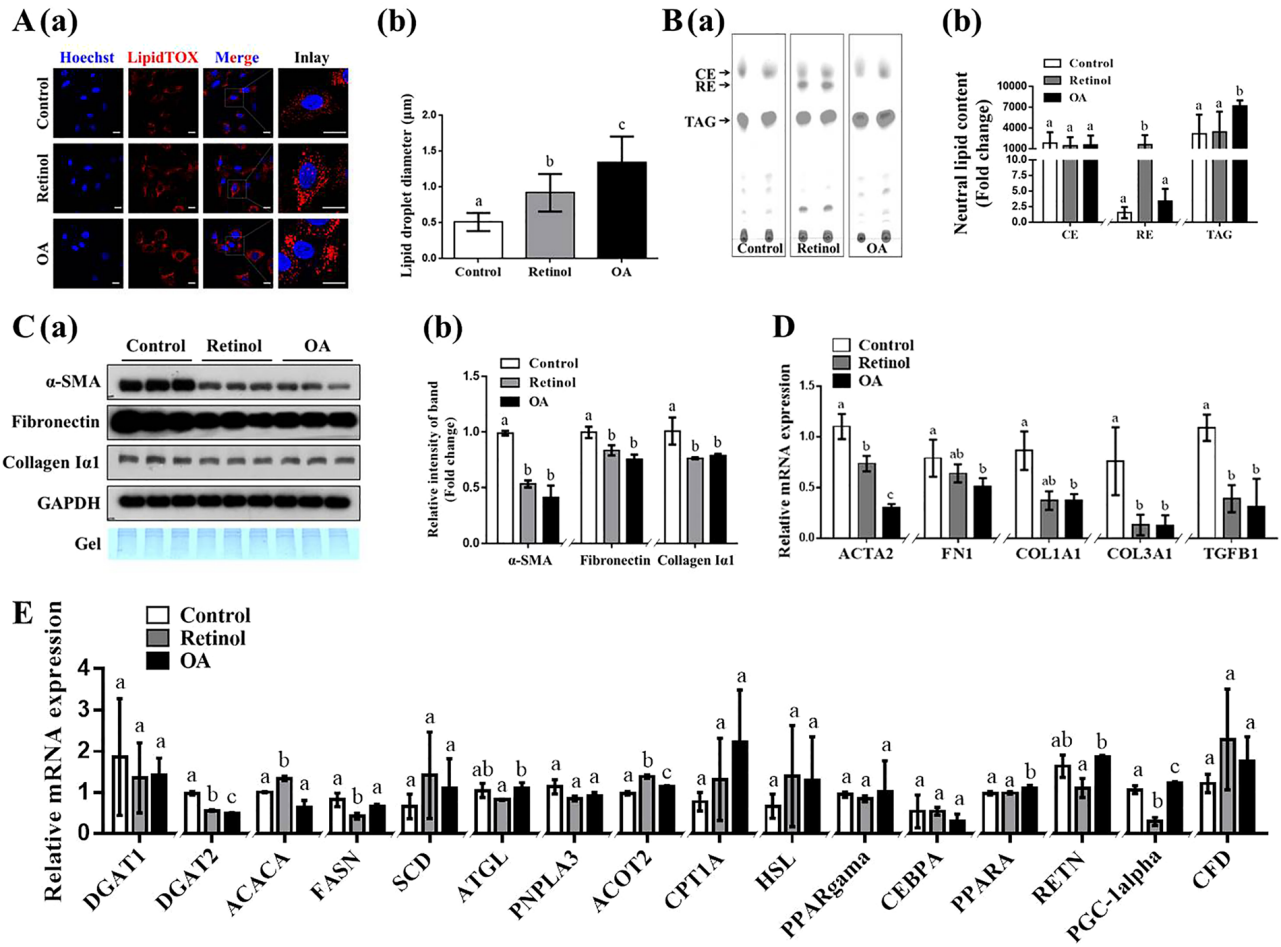
Retinol/oleic acid (OA)-induced enlargement of lipid droplets (LDs) occurs in parallel with the reversion of activated LX-2 cells. (**A**) Retinol/OA-treated LX-2 cells stained with LipidTOX (red) (a) and the diameter of their LDs (b). (**B**) Neutral lipid content and composition (a) and their quantification (b) by thin layer chromatography (TLC). (**C**) Immunoblots (a) and quantification (b) of α-SMA, fibronectin and Collagen Iα1. (**D**) Transcriptional levels of genes associated with hepatic stellate cell (HSC) activation. (**E**) Transcriptional levels of genes involved in lipid metabolism based on RT-qPCR analysis. GAPDH was used as the internal control. Full-length blots are presented in Supplementary Figure 4. Data are presented as the mean ± SD (n = 3) and compared by ANOVA. Significant differences (p < 0.05) are indicated by different letters.

The effects of moderate administration of retinol or OA on the reversion of activated LX-2 cells and the enlargement of their LDs were confirmed by *in vitro* studies. Compared to that in the control group, the intracellular LD diameter in LX-2 cells was 0.4- and 0.8- fold higher in the retinol and OA-treated groups, respectively (Fig. 1A). TLC analysis revealed that TAG content was increased 1.3-fold with OA treatment and that RE was almost exclusively detected in the retinol treatment group (Fig. 1B). Protein and transcription levels of key genes such as *α-SMA (ACTA2)*, *fibronectin (FN1)*, *collagen Iα1 (Col1A1)*, *collagen IIIα1 (Col3A1)*, and *TGFB1*, which reflect HSC activation into myofibroblast-like cells, were compared by immunoblotting and RT-qPCR analyses (Fig. 1C–D). The expression levels of α-SMA, fibronectin and collagen Iα1 were significantly lower with retinol or OA treatment, compared to those in the control group (Fig. 1C). RT-qPCR analyses revealed that transcription levels of *ACTA2* were significantly different between treatments, with the lowest copy numbers occurring with OA treatment, followed by retinol treatment, and the control group (Fig. 1D). The transcription of *Col3A1* and *TGFB1* were remarkably downregulated in response to retinol and OA treatment (Fig. 1D). Transcription levels of key genes associated with lipogenesis (*DGAT1*, *DGAT2*, *ACACA*, *FASN*, and *SCD*), lipolysis (*ATGL*, *PNPLA3*, *ACOT2*, *CPT1A*, and *HSL*) and other genes associated with lipid metabolism (*PPAR-γ*, *CEBPA*, *PPARA*, *RETN*, *PGC-1α*, and *CFD*) were also partly changed in the retinol and OA treatment groups (Fig. 1E). Compared to those in the control group, mRNA levels of *ACOT2* (acyl-coenzyme A thioesterase 2) were significantly higher with retinol and OA treatments, whereas those of *DGAT2* (diacylglycerol O-acyltransferase 2) were significantly lower (Fig. 1E). Significant changes in *ACACA* (acetyl-CoA carboxylase) and *FASN* (fatty acid synthase) were only observed in the retinol-treated group (Fig. 1E). Additionally, increased transcription of *PPARA* (peroxisome proliferator-activated receptor alpha) was observed with OA treatment (Fig. 1E). These results indicated that retinol or OA could sufficiently attenuate HSC activation possibly by modulating lipid metabolism.

### Retinol/OA retard spontaneous activation and loss of LDs in primary mHSCs

**Figure 2:**
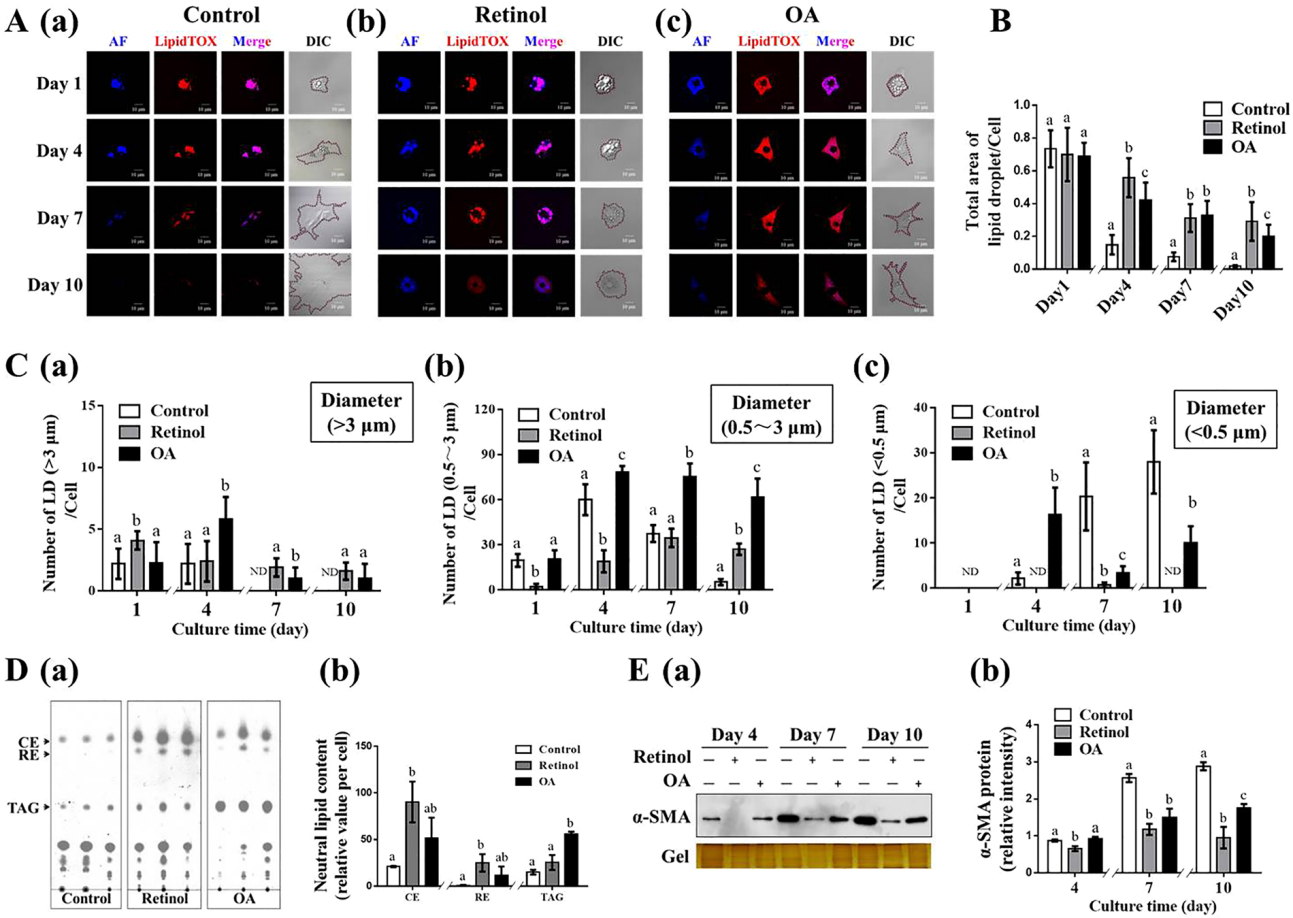
Effects of retinol or oleic acid (OA) on the retardation of spontaneous activation of primary mouse hepatic stellate cells (mHSCs) *in vitro* and their lipid droplets (LDs). (**A**) Dynamics of LDs and associated lipids (blue for retinoids and red for various neutral lipids). (**B**) Total area of LDs per cell. (**C**) Numbers of large, medium, and small LDs per cell. (**D**) Neutral lipid content and composition on day 7. (**E**) Immunoblot analysis of α-SMA. Full-length blots are presented in Supplementary Figure 5. Significant differences (p < 0.05) are indicated by different letters.

Freshly isolated primary mHSCs which undergoes spontaneous activation *in vitro* for mimicing the differentiation of HSCs upon liver injury (Fig. S1), were utilized to explore the effects of retinol and OA on the spontaneous activation of primary mHSCs. The rapid loss of LDs and associated lipids was accompanied by increased expression of α-SMA, fibronectin and collagen Iα1 (Fig. S1C–D). As visualized and quantized in Fig. 2A and B, freshly isolated HSCs (day 1) featured a large number of LDs in the control state, accounting for greater than 70% of total cell area, but the ratio dropped significantly on day 4 and 7, and was barely detected on day 10. The addition of retinol or OA elicited 2.5-fold, 3-fold, and 14-fold increases in the percentage of LD area on day 4, 7, and 10 respectively (Fig. 2A,B). When assessing different-sized LDs, the number of small LDs (<0.5 μm) per cell was increased with the spontaneous activation of primary mHSCs, whereas the number of large LDs (>3 μm) rapidly decreased on day 7 to an undetectable level (Fig. 2C). Compared to that in the control group, the number of large LDs rather than small ones was significantly higher with retinol or OA treatment on day 7 and 10 (Fig. 2C). Correspondingly, the lipid content especially RE and TAG was strongly induced by retinol and OA (Fig. 2D). In addition, the delay in lipid loss, the expression of α-SMA, considered a common marker of stellate cell activation, was significantly suppressed with the addition of retinol or OA, compared to that in control conditions (Fig. 2E). Taken together, cell line and primary cell experiments suggest that the suppressive effects of retinol and OA on HSC activation could be due to the changes in LDs.

### Proteomic analysis reveals differentially expressed LD proteins during the reversion of activated LX-2 cells

To comprehensively reveal potential LD-associated proteins that play roles in regulating HSC activation, LD isolation and comparative proteomic analysis were performed. Human stellate cell LDs were purified according to a previously published method^33^. The main bands of the LD profile identified by mass spectrometry were compared with other published LD proteomes (Fig. S3B).

**Figure 3:**
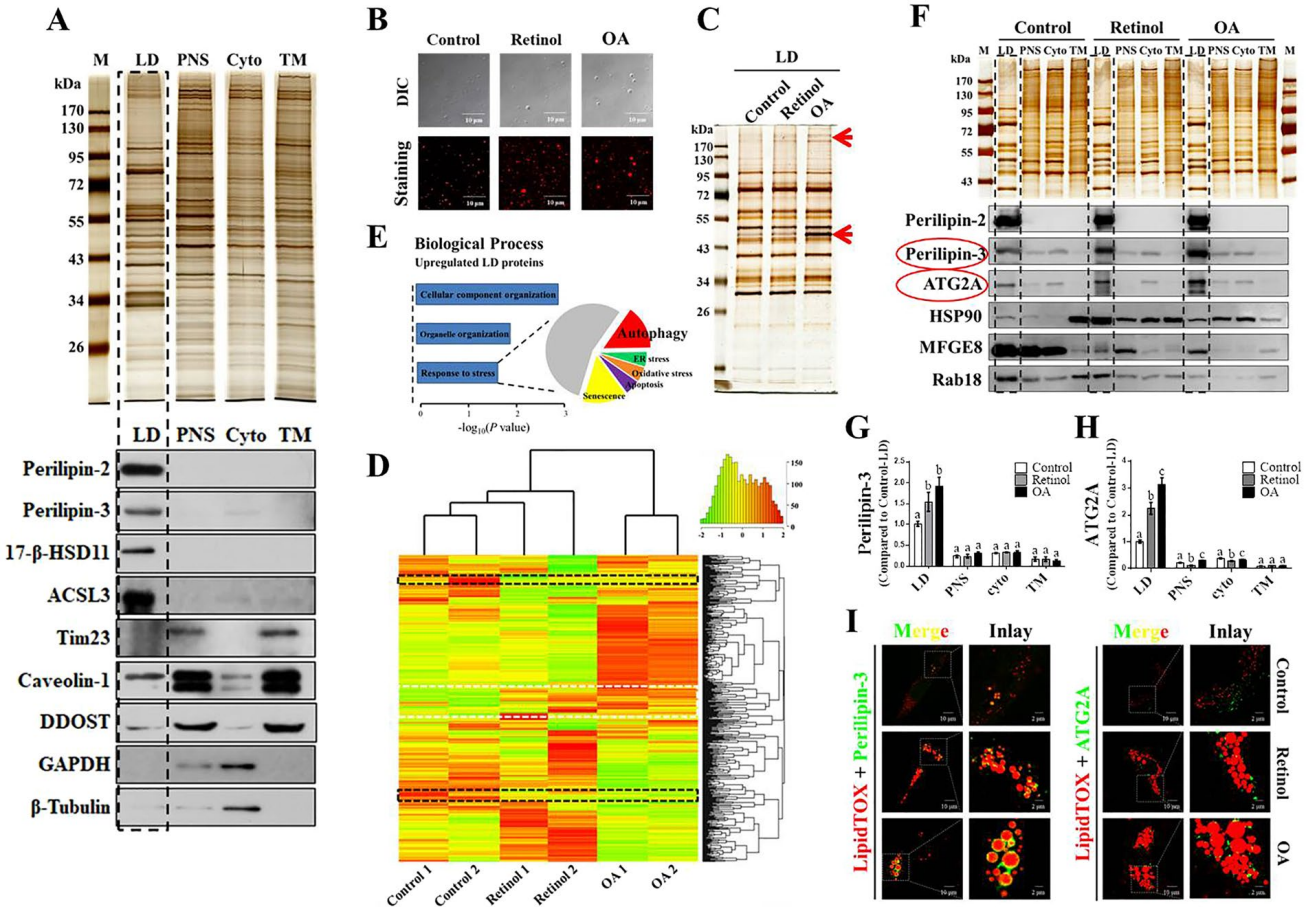
Proteomic analysis reveals differentially expressed lipid droplet (LD) proteins during the reversion of activated LX-2 cells induced by retinol or oleic acid (OA). (**A**) Silver stained SDS-PAGE gel and immunoblots of various cellular location markers for the equal-loading proteins from isolated LDs, postnuclear supernatant (PNS), cytosol (Cyto), and total membrane (TM) fractions. (**B**) Isolated LDs in retinol or OA treatment observed by confocal microscopy. (**C**) Silver stained gel of LD proteins and some distinct protein bands indicated by red arrows. (**D**) Heat map visualization of LD protein composition from TMT-based mass spectrometry and proteins commonly upregulated (white dashed line) and downregulated (dark dashed line) upon retinol or OA treatment. (**E**) Biological processes associated with upregulated proteins based on the KEGG web database. (**F**–**H**) Validation of changes observed in quantitative proteome; six LD proteins (perilipin-2, perilipin-3, ATG2A, HSP90, MFGE8, and Rab18) were assessed by immunoblotting, and associated quantification is shown. (**I**) Localization of perilipin-3 and ATG2A on LDs based on immunofluorescence. Full-length blots are presented in Supplementary Figure 6. Significant differences (p < 0.05) are indicated by different letters.

To assess the quality of isolated LDs, the protein profile of LDs, based on silver staining gels, was quite different from that in other cellular fractions including post nuclear supernatant (PNS), cytosol (Cyto), and total membrane (TM) (Fig. 3A). Furthermore, subcellular organelle/compartment-specific markers were tested in the four equal-loading fraction, including four LD-specific markers (perlipin-2, perlipin-3, 17-β-HSD11, and ACSL3), one mitochondrial marker (Tim23), one plasma membrane marker (caveolin-1), one endoplasmic reticulum (ER) marker (DDOST), and two cytoplasmic markers (GAPDH and β-Tubulin) (Fig. 3A). Significantly, LD-specific marker proteins were selectively enriched in the LD fraction, indicating the high purity of LDs (Fig. 3A).

Based on this established technique for LD isolation, three independent LD preparations in each treatment group were carried out (Fig. S3A). The sizes of isolated LDs from the three groups, as visualized by LipidTOX staining (Fig. 3B) and measured by a Delsa Nano C particle analyzer (Fig. S2B), conformed with those observed *in vivo* (Fig. 1A). The LD protein profiles, as assessed by silver staining, showed some distinct protein bands (red arrows) between the groups (Fig. 3C). To rapidly identify and accurately quantify hundreds of LD proteins, TMT-based mass spectrometry was utilized to provide an overview of the dynamic changes during the reversion of activated LX-2 cells (Fig. S2A). In total, 496 LD proteins were detected by quantitative proteomic analysis (Fig. S2C; Table S3). Hierarchical cluster analysis revealed that protein expression patterns varied between treatments (Fig. 3D). Among them, 50 proteins were consistently upregulated (outlined with a white dashed line) and 28 were downregulated (outlined with a dark dashed line) (Fig. 3D; Table S4). Changes in the expression of 16 proteins including LD-resident proteins (perlipin-2, perlipin-3) and lipid metabolism-related proteins (e.g. ACSL3, ACSL4, CGI-58, and 17-β-HSD11) were also validated by immunoblot analyses (Fig. 3F–H; Fig. S3C). Among these proteins, Rab18 was decreased in all analyzed cellular fractions upon treatment with retinol or OA (Fig. 3C–D), which was consistent with other studies^23^. Most strikingly, significant aggregation of perilipin-3 (molecular weight: 48 kDa) and ATG2A (molecular weight: 212 kDa) on retinol/OA-treated LDs was detected (Fig. 3C; Fig. F-H), which was further confirmed by immunofluorescence assays (Fig. 3I). Especially, the effects of retinol or OA on the expression pattern of ATG2A varied among different cellular fractions, with increased expression observed in LDs but decreased expression noted in the cytosol (Fig. 3H), which suggests that LDs might recruit ATG2A during the reversion of activated HSCs.

### Inhibition of ATG2A facilitates the transition of HSCs and their LDs

**Figure 4:**
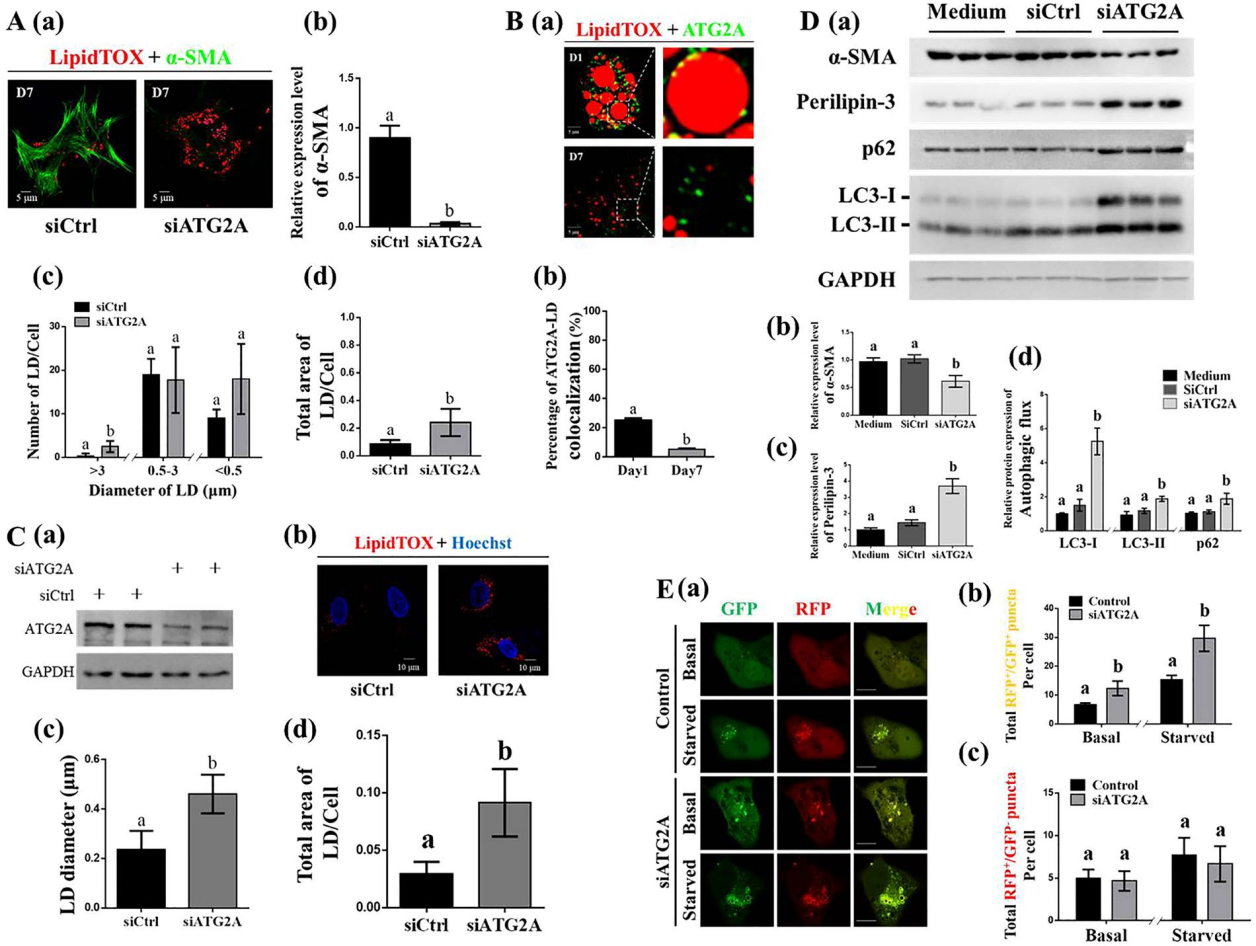
Involvement of ATG2A in hepatic stellate cell (HSC) activation. (**A**) Effects of ATG2A knockdown on the spontaneous activation of primary HSCs (a) including α-SMA expression (b), number of large, medium, and small LDs per cell (c), and total area of LDs per cell (d). (**B**) ATG2A-LD co-localization in quiescent (freshly isolated on day 1) and activated (cultured *in vitro* on day 7) primary mHSCs. (**C**) Effects of siRNA-mediated inhibition of ATG2A (a) on the LD morphology (b–d) of activated LX-2 cells. (**D**) Protein levels associated with activation (α-SMA) (b), LD scaffold proteins (perilipin-3) (c), and autophagic flux (LC3 and p62) (d) in ATG2A-knockdown LX2 cells (a). (**E**) Representative images and quantification of early autophagosomes (yellow dots showing overlapping GFP and RFP puncta) and late autolysosomes (red dots only; RFP puncta); LX-2 cells transfected with negative siRNAs or siATG2A and co-transfected with the GFP-RFP-hLC3 plasmid were incubated in complete or starvation media for 4 hours. Full-length blots are presented in Supplementary Figure 7. Data were presented as mean ± SD and compared by ANOVA. Significant differences (p < 0.05) are indicated by different letters.

To explore the role of ATG2A in the transition of HSCs from quiescence to activation or vice versa, we used siRNA to decrease ATG2A expression in primary mHSCs and LX-2 cells. Immunoblot analyses confirmed the efficient depletion of ATG2A in LX-2 cells (Fig. 4C), from which sufficient protein extraction was feasible. ATG2A knockdown resulted in lower expression of α-SMA, suggesting that ATG2A inhibition delayed the spontaneous activation of primary mHSCs (Fig. 4A). An increased number of large LDs (>3 μm) and a higher LD area per cell ratio were found in the inhibited cells, compared to those in control cells (Fig. 4A). Interestingly, a larger fraction (>20%) of ATG2A was detected as tiny punctate structures around LDs in quiescent mHSCs. In contrast, less than 5% of ATG2A colocalized with LDs in activated mHSCs (Fig. 4B). The LD diameter and the ratio of LD area per cell in LX-2 cells were higher in the ATG2A-knockdown cells, compared to those in the control condition (Fig. 4C). As expected, as a ubiquitously expressed LD resident protein^38^, perilipin-3 which was shown to coat the LDs of stellate cell (Fig. 3I; Fig. S1B), and was significantly increased in lipid-rich cells induced by ATG2A depletion (Fig. 4D). In addition, the expression of α-SMA was lower in ATG2A-knockdown LX2 cells (Fig. 4D). The expression of p62 and LC3 which were used to monitor the autophagic flux were analyzed by immunoblotting and morphological tracing. Increased expression of p62 and LC3-II were observed in the ATG2A-depleted LX-2 cells (Fig. 4D). Morphological tracing analysis using fluorescent-tagged LC3 (GFP-RFP-hLC3) further confirmed a block in autophagic degradation in ATG2A-depleted cells as evidenced by an increase of yellow puncta (immature/completed autophagosomes) and a comparable level of red puncta (autolysosomes) (Fig. 4E). Thus, all results demonstrated that the pivotal role of ATG2A in mediating cell activation and autophagy in HSCs.

## Discussion

The reversion of the activated LX-2 human hepatic stellate cell line, with retinol or OA treatment, was accompanied by LD enlargement. To date, clinical studies and animal experiments have proven the efficiency of retinol and OA for the prevention or treatment of liver fibrosis^10,13,14^. In the present study, the inhibitory effects of OA on HSC activation were observed in both LX-2 cells and primary mHSCs, in accordance with previous reports^14,39^. In addition, the suppressive effects of retinol alone on collagen production and HSC proliferation were previously only reported for primary HSCs, and not for HSC cell lines^40,41^. Nevertheless, retinol treatment supplemented with other reagents such as insulin or palmitic acid significantly inhibits the expression of α-SMA in primary rat HSCs and LX-2 cells^16,17^. Moreover, retinoic acid, a metabolite of retinol, has significant inhibitory effects on α-SMA, COL1A1, and MMP2 in primary human HSCs and LX-2 cells^42–44^. Here, the reversion of activated LX-2 cells was observed with retinol treatment after cell synchronization by serum starvation, which is well known to induce autophagy in many types of cultured cell^45–47^. Thus, the reversion of LX2 cells is possibly due to a synergistic effect of starvation and retinol, in agreement with recent findings that autophagy is related to HSC activation^48,49^.

Through quantitative comparative proteomic analysis, 496 LD proteins were detected in LDs purified from LX-2 cells. Among them, 77 were associated with the ER (Fig. S2D), in agreement with the proposed hypothesis that the formation of LDs occurs from the ER^50^. The magnitude of LD proteomes was also comparable to that reported in other studies^21,22^. Heat map analysis suggested that both retinol and OA triggered shifts in LD proteins. Most strikingly, 78 LD proteins were similarly changed in both retinol- and OA-treated reverting LX-2 cells. Among these, levels of Rab18, which are associated with HSC activation/reversion, were in accordance with those reported in previous studies^23^. However, the roles of other proteins in the transition of HSCs remain to be explored. These proteins serve as potential targets to understand the complex mechanisms associated with HSC activation and thus the amelioration of liver fibrosis.

**Figure 5:**
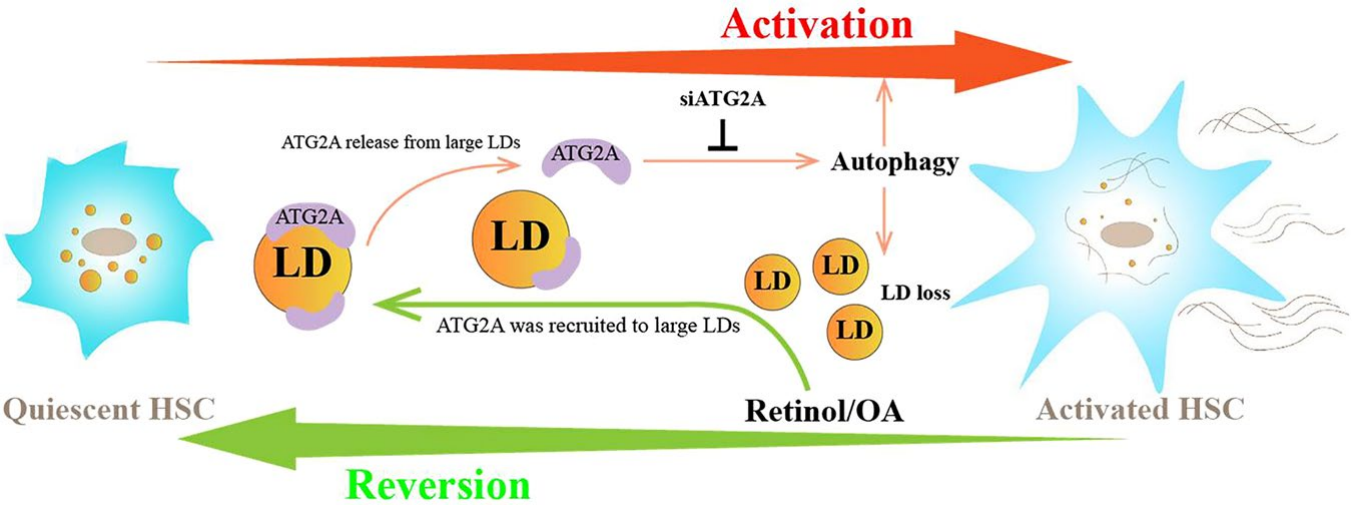
Schematic of the positive feedback model for the regulation of lipid droplets (LDs) and hepatic stellate cell (HSC) activation by ATG2A.

Fifty proteins that were consistently upregulated with retinol and OA treatments were annotated based on biological function and analyzed using the KEGG database. This revealed that the stress response was significantly enriched among upregulated proteins (Fig. 3E), and this included autophagy-related proteins (ATG2A, WDR45, and RUVBL1, among others). Human ATG2A, which is essential for autophagosome formation^51,52^, was previously reported to localize to the LDs of human cell lines including U2OS, Hela, and G361^51,53^. In the present study, aggregation of ATG2A was found to be increased around the large LDs of retinol- or OA-induced reverted HSCs. In contrast, the relocation of ATG2A from the LDs to other cytoplasmic structures was observed along with the activation of primary HSCs (Fig. 4B). Considering the roles of ATG2A in autophagy, which is required for HSC activation^54^, the release of this protein from LDs might trigger HSC activation. Indeed, robust inhibition of α-SMA in both primary mouse HSCs and the human LX-2 HSC line was observed upon ATG2A knockdown, suggesting that ATG2A is a key mediator of HSC activation. Accordingly, the growth of LDs and the increase of their resident protein perilipin-3 in siATG2A-treated LX2 cells were evident based on microscopy and immunoblotting, respectively. These findings provide the first evidence that ATG2A plays a vital role linking autophagy to HSC activation. We propose a positive feedback model of ATG2A during the regulation of LDs and HSC activation (Fig. 5). LDs could serve as reservoirs for ATG2A in quiescent HSCs, and the release of ATG2A from LDs might trigger autophagy, which would result in the degradation of LDs to fuel HSC activation (Fig. 5). In contrast, the enlarged LDs could result in the recruitment of ATG2A to downregulate autophagy and reverse the activation of HSCs (Fig. 5). Together, the current study provides insights into the mechanisms underlying autophagy and HSC activation/reversion via ATG2A, and suggests a potential therapeutic benefit for mitigating liver fibrosis by targeting this protein. However, more basic biomedical studies and even clinical studies are still needed to confirm the effects of ATG2A depletion on liver fibrosis treatment.

## Electronic supplementary material

Supplemental Information file

Dataset 1
