# Author Correction: *In vitro* inhibition of hepatic stellate cell activation by the autophagy-related lipid droplet protein ATG2A

**DOI:** 10.1038/s41598-018-32560-6

**Published:** 2018-09-26

**Authors:** Yun Hong, Sirui Li, Jifeng Wang, Youming Li

**Affiliations:** 1 First Affiliated Hospital, School of Medicine, Zhejiang University, Hangzhou, China; 2 Institute of Biophysics, Chinese Academy of Sciences, Beijing, China; 3 University of Chinese Academy of Sciences, Beijing, China

Correction to: *Scientific Reports* 10.1038/s41598-018-27686-6, published online 18 June 2018

In the original version of this Article, Youming Li was omitted as a corresponding author. Correspondence and request for materials should also be addressed to zlym@zju.edu.cn. This error has now been corrected in the HTML and PDF versions of the Article and in the accompanying Supplemental Information file.

