# Supplementary material for: In vitro inhibition of hepatic stellate cell activation by the autophagy-related lipid droplet protein ATG2A
Source: Sci Rep. 2018 Jun 18;8:9232. doi: 10.1038/s41598-018-27686-6 (PMC6006255; doi:10.1038/s41598-018-27686-6)
Supplement: Supplementary file 1 — Supplemental Information file [file 41598_2018_27686_MOESM1_ESM.pdf]

Supplementary Information file

In vitro inhibition of hepatic stellate cell activation by the autophagy-related lipid droplet protein ATG2A

Yun Hong*, Sirui Li, Jifeng Wang, Youming Li*

***: To whom correspondence should be addressed:**
Yun Hong
E-mail: yunhong@zju.edu.cn
Youming Li
E-mail: zlym@zju.edu.cn

Supplemental Figure 1:

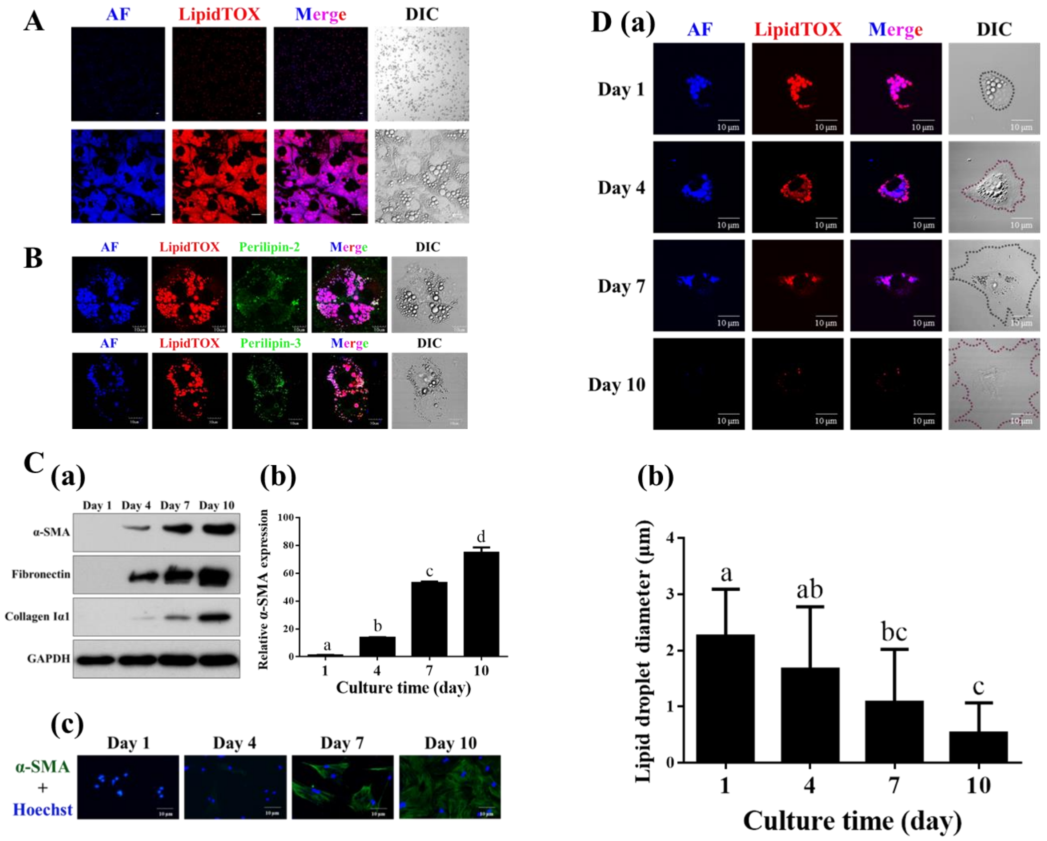

Supplemental Fig. 1. Stable, established model of *in vitro* primary mouse hepatic stellate cell (mHSC) activation. (A) Primary HSCs isolated from C57BL/6 mice. Intracellular LDs with retinoid content exhibited transient autofluorescence (AF, blue), whereas other neutral lipids (triacylglycerol, cholesterol esters, etc.) were stained by LipidTOX (red). (B) Immunofluorescence of LD scaffold proteins (perilipin-2 and perilipin-3). (C) Immunoblots for α-SMA, fibronectin, and collagen Iα1 (a). Immunoblot quantification (b) and immunofluorescence (c) for α-SMA. Full-length blots are presented in Supplemental Figure 8. (D) Dynamic morphology of primary HSCs cultured on dishes on days 1, 4, 7 and 10 (a). Quantification of LD diameter (b). Significant differences ($p < 0.05$) are indicated by different letters.

Supplemental Figure 2:

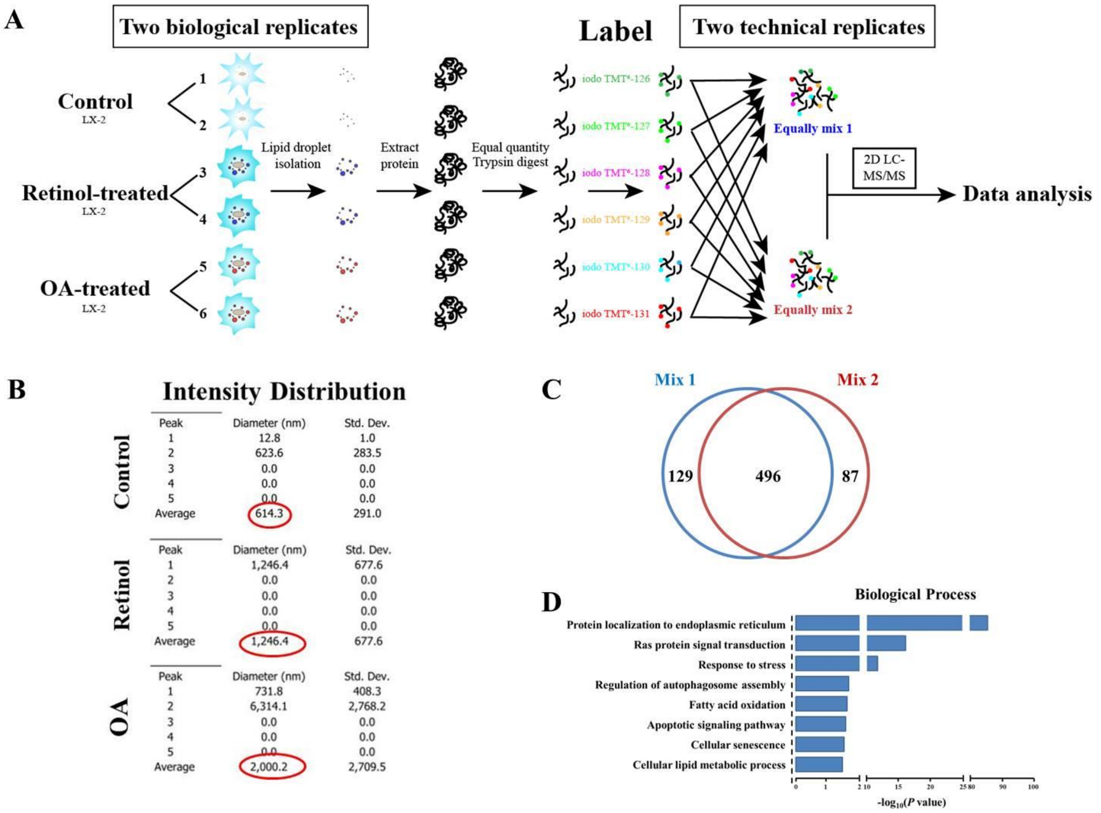

Peak	Diameter (nm)	Std. Dev.
**Control**		
1	12.8	1.0
2	623.6	283.5
3	0.0	0.0
4	0.0	0.0
5	0.0	0.0
Average	614.3	291.0
**Retinol**		
1	1,246.4	677.6
2	0.0	0.0
3	0.0	0.0
4	0.0	0.0
5	0.0	0.0
Average	1,246.4	677.6
**OA**		
1	731.8	408.3
2	6,314.1	2,768.2
3	0.0	0.0
4	0.0	0.0
5	0.0	0.0
Average	2,000.2	2,709.5

Supplemental Fig. 2. TMT-based quantitative proteomic analysis of isolated lipid droplets (LDs). (A) Flow chart of experimental scheme. (B) Isolated LDs dimensioned by a Delsa Nano C particle analyzer. (C) Venn diagram of identified LD proteins based on two technical replicates (blue and red). (D) Biological processes associated with the 496 LD proteins based on the KEGG web database.

Supplemental Figure 3:

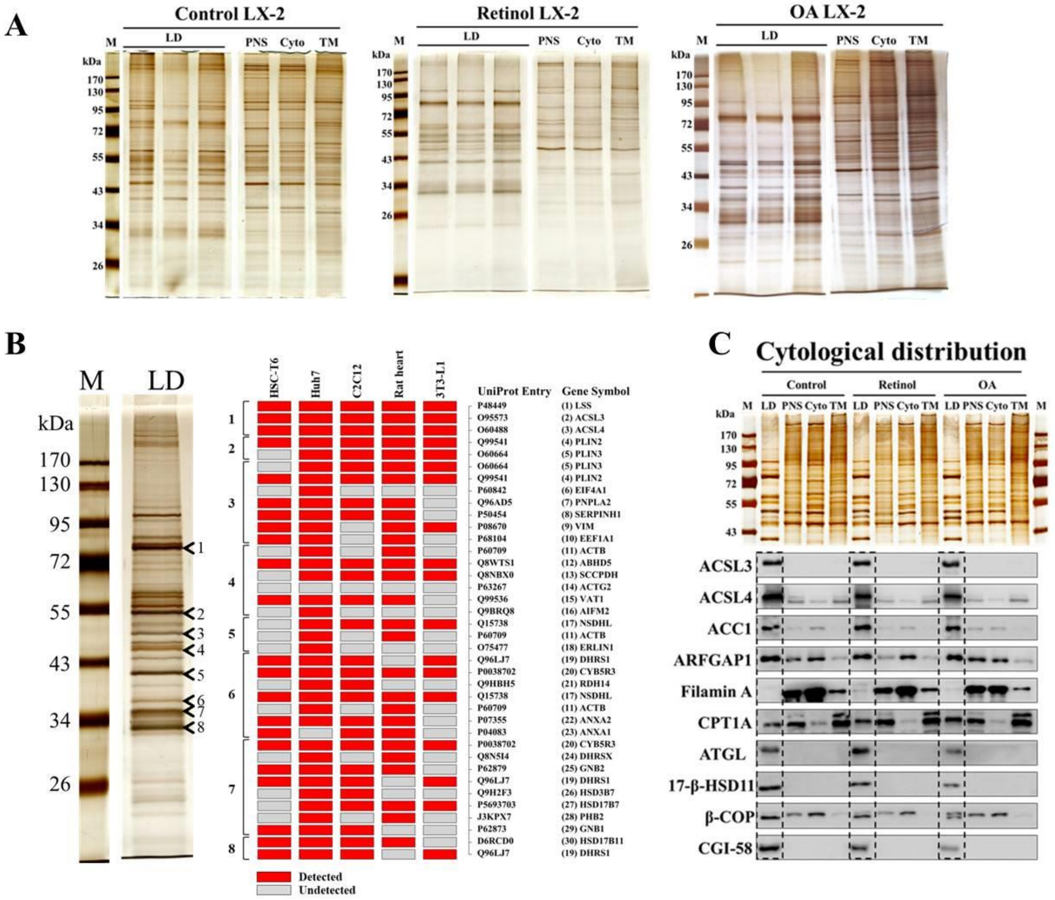

Supplemental Fig. 3. High quality isolation of lipid droplets (LDs) from LX-2 cells. (A) Silver stained gels with LD proteins purified in triplicate for each group. (B) Main bands with arrows from LD protein lane were excised for mass spectrometry analysis and compared with other published proteomes. (C) Immunoblots of some LD proteins identified by mass spectrometry. Full-length blots are presented in Supplemental Figure 9.

Supplemental Figure 4: Full western blot of Figure 1C

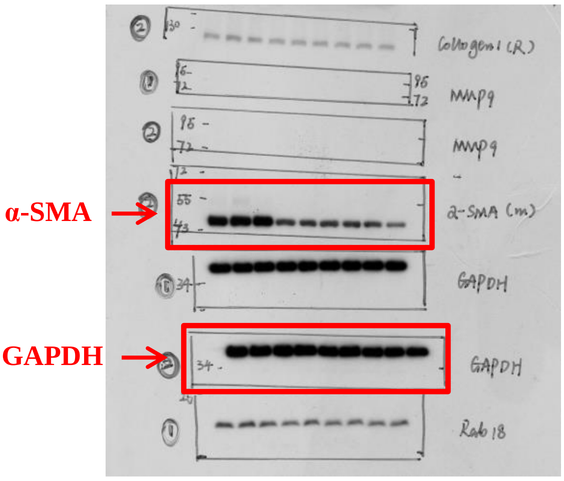

Exposure time 5 sec

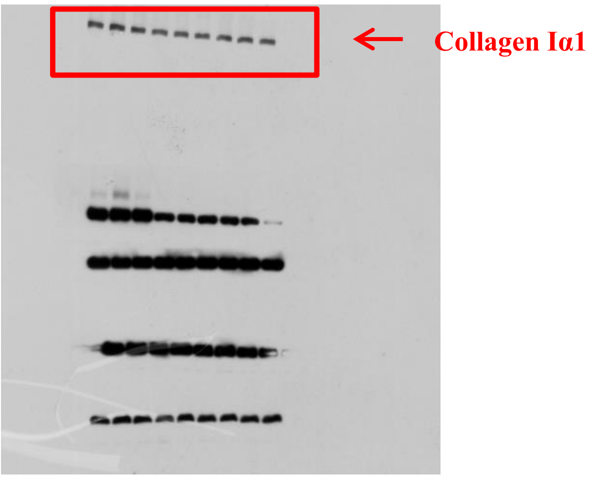

Exposure time 15 sec

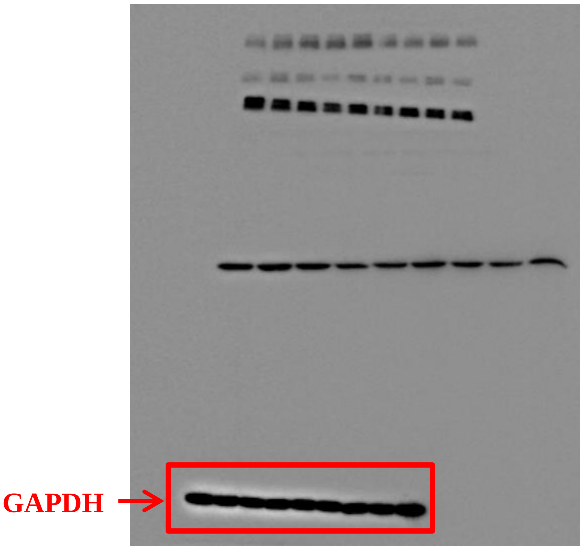

Exposure time 10 sec

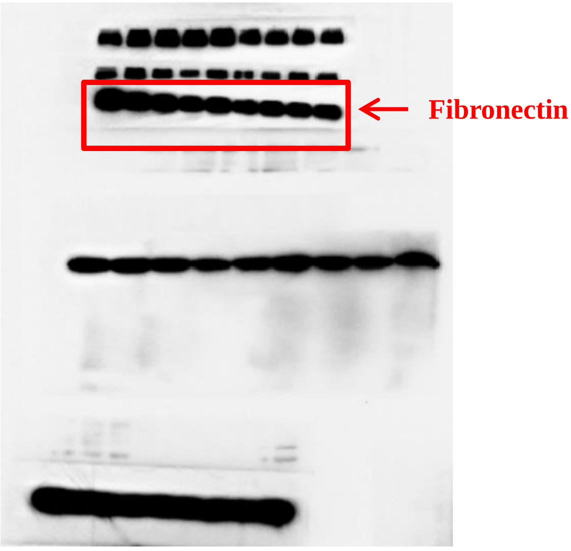

Exposure time 20 sec

Supplemental Figure 5: Full western blot of Figure 2E

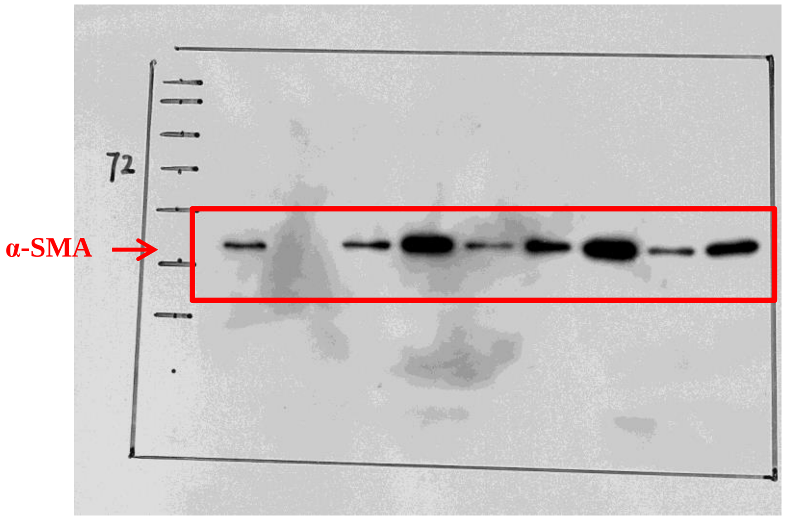

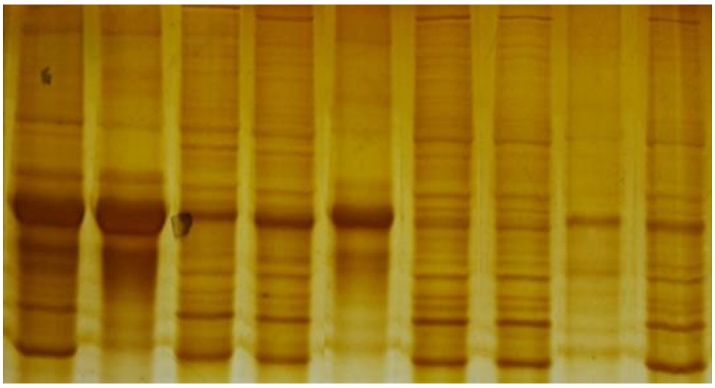

The gel for silver staining after transferring PVDF membrane

Supplemental Figure 6: Full western blot of Figure 3A

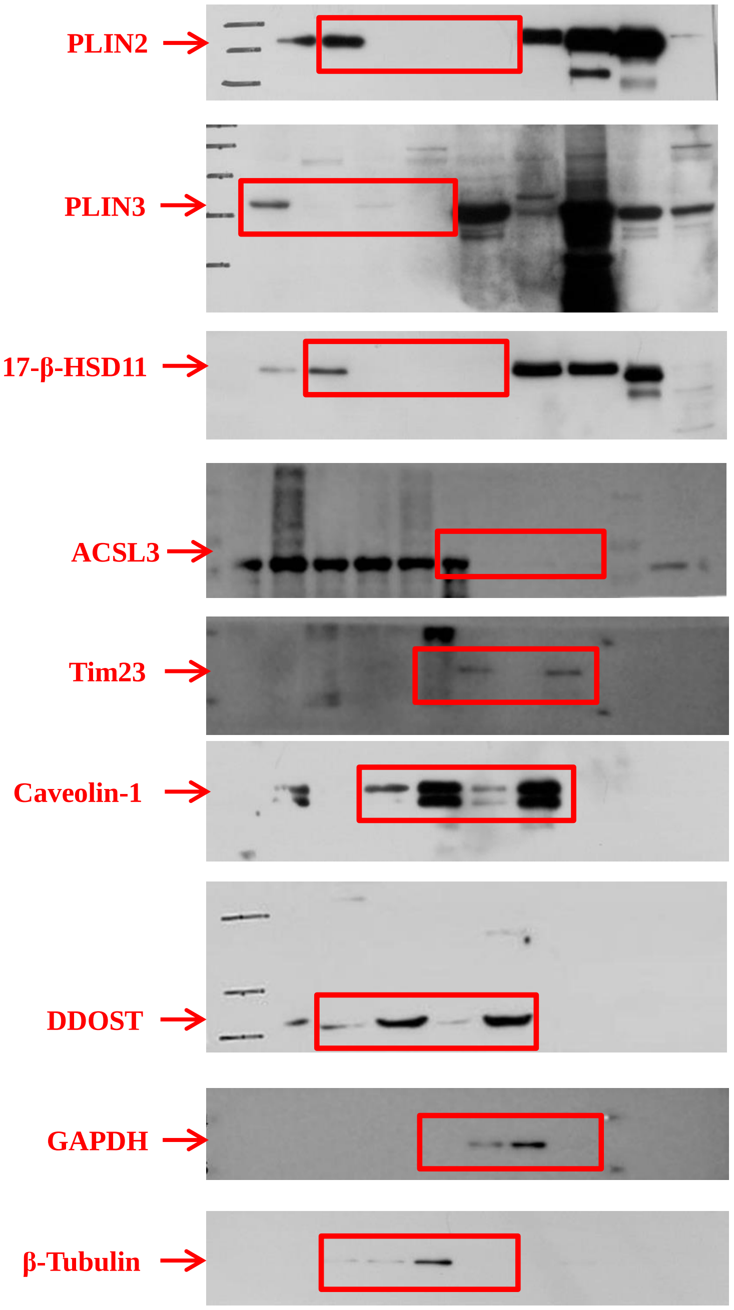

Full western blot of Figure 3F

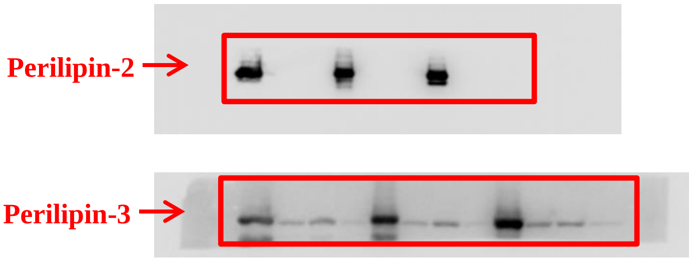

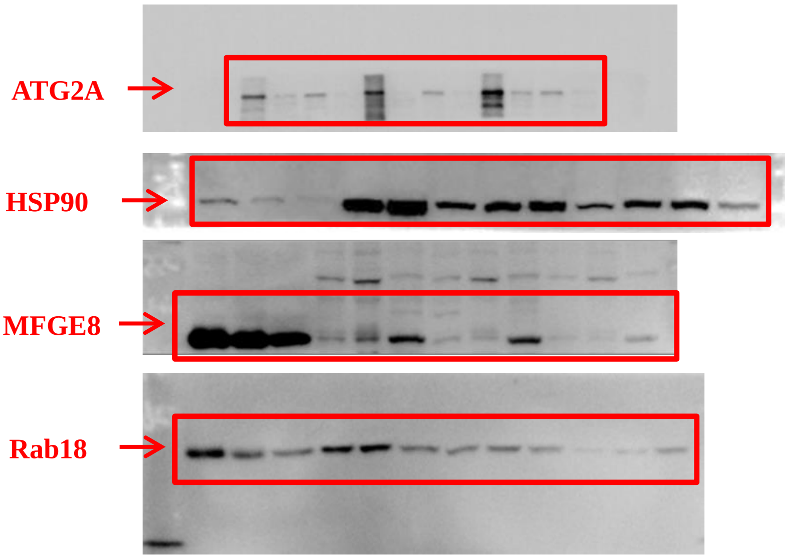
ATG2A
HSP90
MFGE8
Rab18

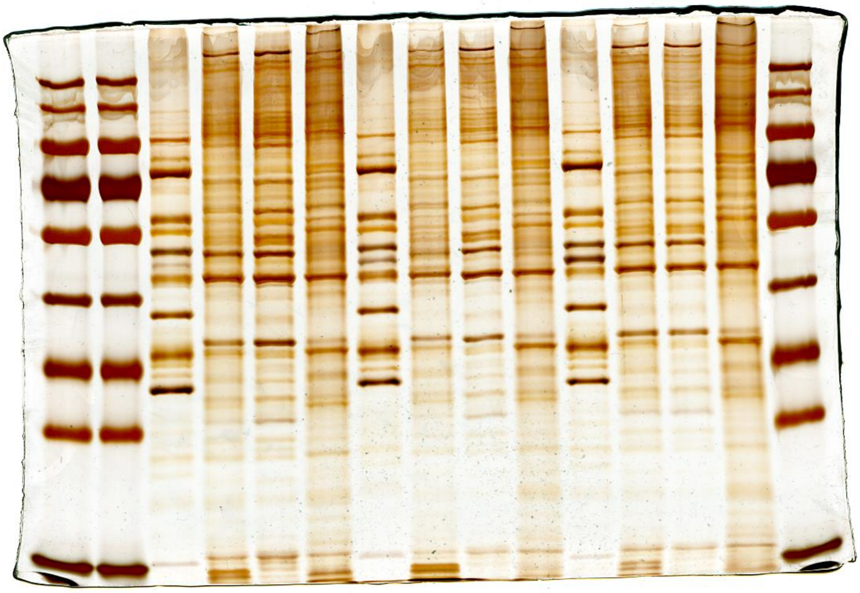

Supplemental Figure 7: Full western blot of Figure 4C and D

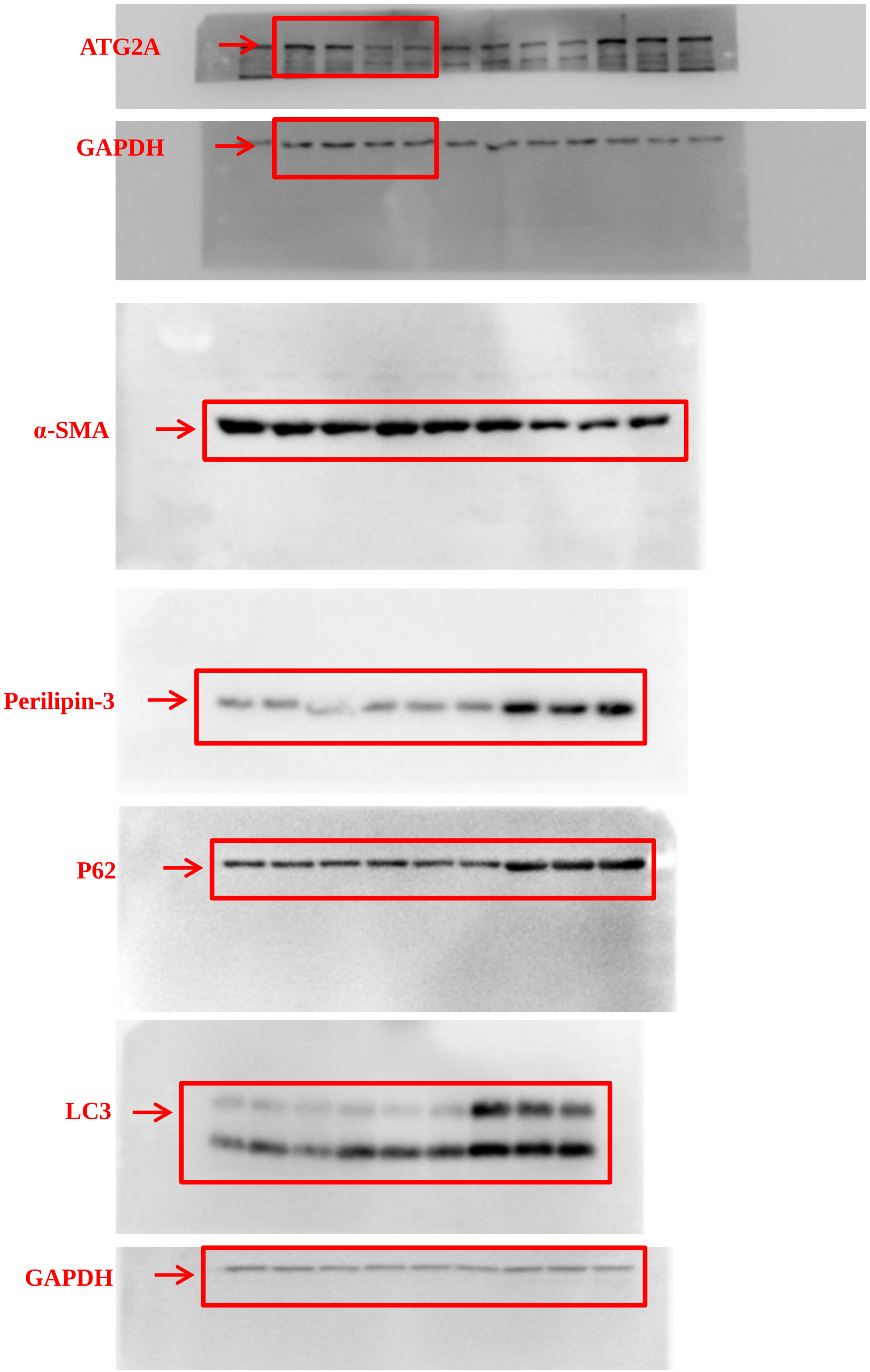

Supplemental Figure 8: Full western blot of Supplemental Figure 1C

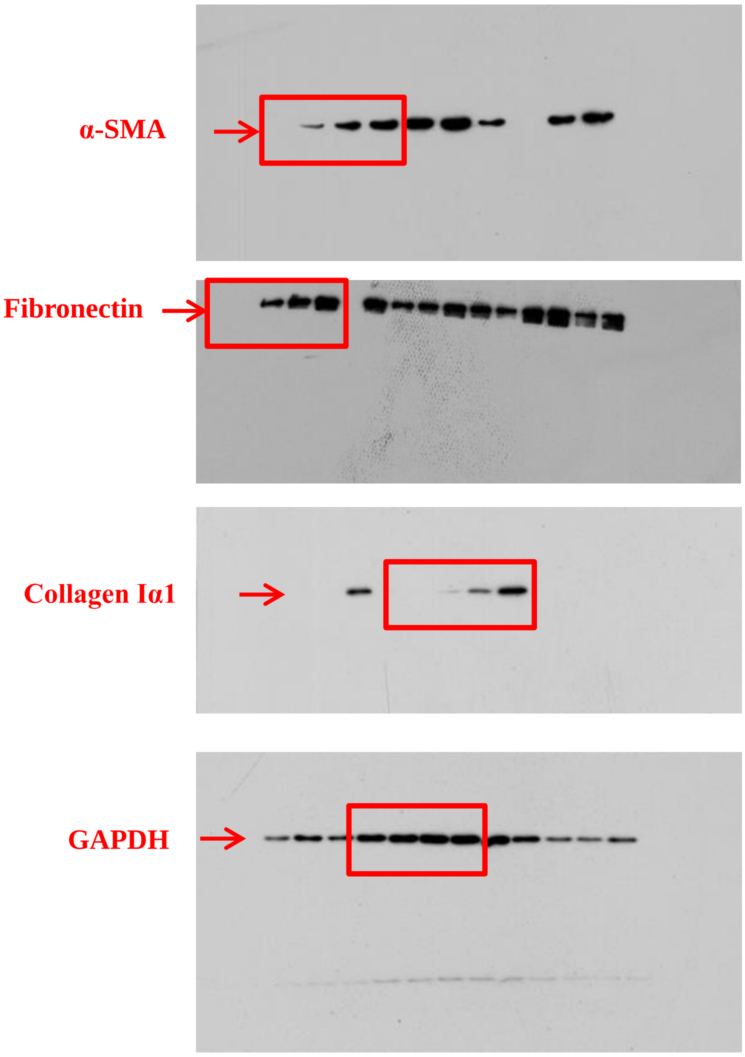

Supplemental Figure 9: Full western blot of Supplemental Figure 3C

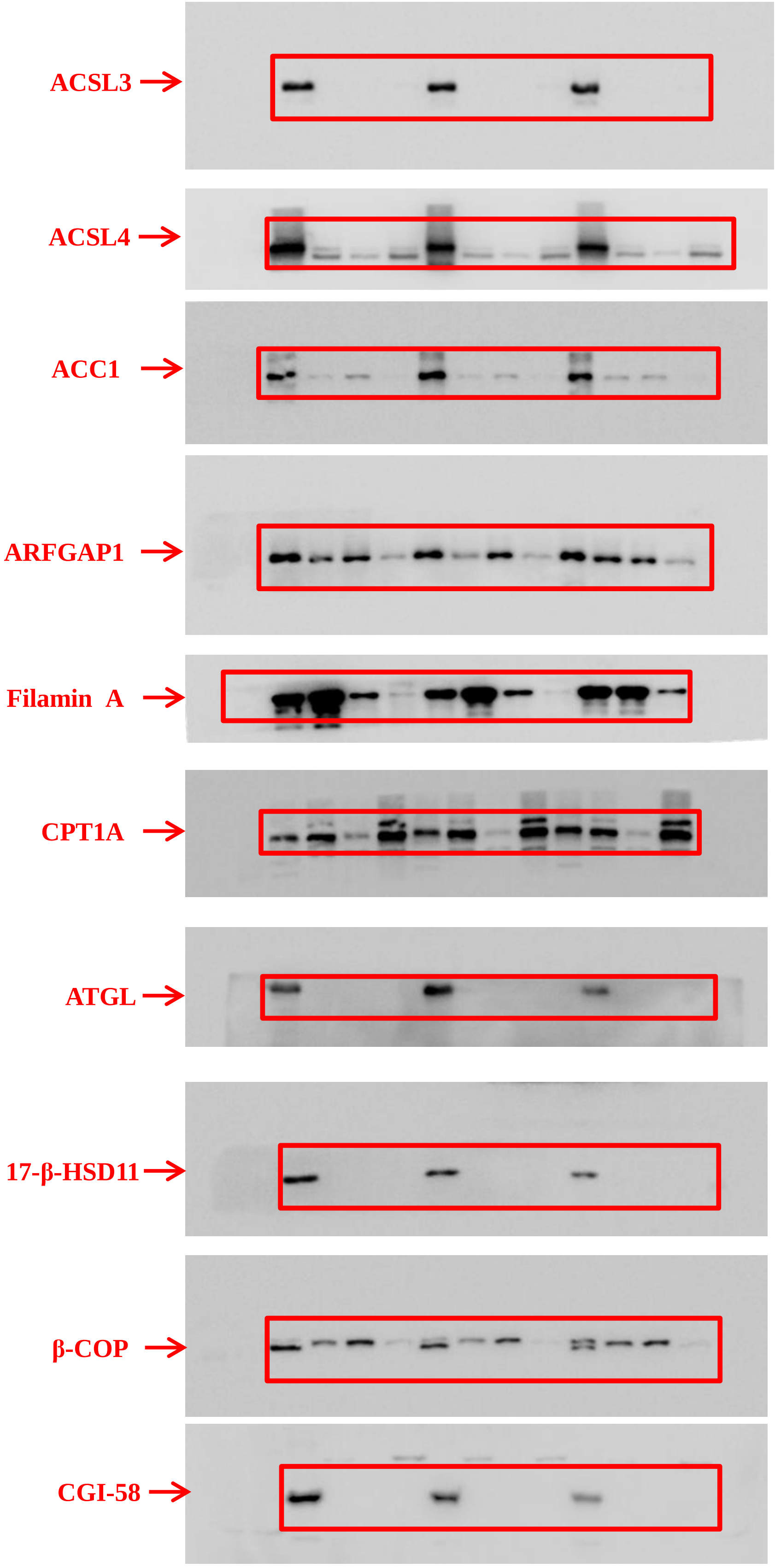

Supplemental Table 1. Key reagents used in this study

REAGENT or RESOURCE	SOURCE	IDENTIFIER
Antibodies (Dilution)		
PLIN2 (1:2000 for WB)	abcam, UK Limited	ab108323
PLIN3 (1:5000 for WB 1:200 for IF)	abcam, UK Limited	ab138593
α-SMA (1:1000 for WB 1:200 for IF)	abcam, UK Limited	ab5694
Fibronectin (1:1000 for WB)	abcam, UK Limited	ab2413
collagen Iα1 (1:500 for WB)	RayBiotech, USA	P02452
GAPDH (1:2000 for WB)	Millipore, Bedford, MA, USA	CB1001
ATG2A (1:500 for WB 1:200 for IF)	MBL Life Science, Japan	PD041
HSP90 (1:1000 for WB)	abcam, UK Limited	ab13492
MFGE8 (1:1000 for WB)	ABclonal Biotechnology, USA	A12322
Rab18 (1:1000 for WB)	Millipore, Bedford, MA, USA	552126
p62 (1:1000 for WB)	BD Transduction Laboratories	610833
LC3 (1:500 for WB)	Proteintech, USA	12135-1-AP
ACSL3 (1:1000 for WB)	Proteintech, USA	20710-1-AP
ACSL4 (1:1000 for WB)	ABclonal Biotechnology, USA	A6826
ACC1 (1:1000 for WB)	Upstate Biotechnology, USA	04-322
ARFGAP1 (1:1000 for WB)	ABclonal Biotechnology, USA	A7118
Filamin A (1:1000 for WB)	Bioworld Technology, USA	BS1128
CPT1A (1:1000 for WB)	Bioworld Technology, USA	BS7744
ATGL (1:1000 for WB)	Cell signaling, MA, USA	2138
17-β-HSD11 (1:1000 for WB)	abcam, UK Limited	ab136109
β-COP (1:1000 for WB)	ABclonal Biotechnology, USA	A10485
CGI-58 (1:1000 for WB)	ABclonal Biotechnology, USA	A7592
Tim23 (1:1000 for WB)	BD Transduction Laboratories	611223
Caveolin-1 (1:1000 for WB)	BD Transduction Laboratories	610060
DDOST (1:1000 for WB)	abcam, UK Limited	ab65533
β-Tubulin (1:2000 for WB)	Cell signaling, MA, USA	2146
Chemicals and Critical Commercial Assays		
Retinol	SIGMA	17772
Oleic acid	SIGMA	O1008
TRIzol Reagent	Invitrogen	T9424
Reverse Transcriptase	Invitrogen	18080-051
LipidTOX Red	Thermo Scientific	H34476
DAPI	SIGMA	D9542
ECL	PerkinElmer	203-13261
OptiPrep Density Gradient Medium	SIGMA	D1556
DNAseI	SIGMA	AMPD1
Pronase E	SIGMA	P5147
Collagenase IV	GIBCO	17104019
Experimental Models: Cell Lines		

LX-2	Millipore, Bedford, MA, USA	SCC064
**Oligonucleotides**		
human ATG2A siRNA (sense: 5'-GCUACUUGCUGCACCACUATT-3', antisense: 5'-UAGUGGUGCAGCAAGUAGCTT-3')	GenePharma	N/A
mouse ATG2A siRNA (sense: 5'-GCCUCAUGCAUGACCACAATT-3', antisense: 5'- UUGUGGUCAUGCAUGAGGCTT-3')	GenePharma	N/A

Supplemental Table 2. Primers used in this study

PRIMERS GENE (HUMAN)	FORWARD SEQUENCE (5'→3')	Tm-Forward primer	REVERSE SEQUENCE (5'→3')	Tm-Reverse primer	Reference
FN1	GCACTACTGGCCAGTCCTACAACCA	66	CGGGAATCTTCTCTGTCAGCCTGTA	66	[1]
TGFB1	CAACGAAATCTATGACAAGTTCAAGCAG	65	CTTCTCGGAGCTCTGATGTG	55	[2]
COL3A1	CTCCTGGGATTAATGGTAGT	51	CCAGGAGCTCCAGGAAT	52	[3]
COL1A1	AACCAAGGCTGCAACCTGGA	63	GGCTGAGTAGGGTACACGCAGG	64	[4]
ACTA2	AGCGTGGCTATTCCTTCGT	57	CTCATTTTCAAAGTCCAGAGCTACA	60	[5]
DGAT1	GCTTCAGCAACTACCGTGGCAT	57	CCTTCAGGAACAGAGAAACCACC	59	
DGAT2	ATAAAGTGTTGCGCTCCGGG	57	CGCGAGAGAAACAGCCTAGA	57	
ACACA	TTCACTCCACCTTGTCAGCGGA	65	GTCAGAGAAGCAGCCCATCACT	61	[6-8]
FASN	TTCTACGGCTCCACGCTCTTCC	66	GAAGAGTCTTCGTCAGCCAGGA	61	[8]
SCD	GCAAACACCCAGCTGTCAAA	55	GCCAGGTTTGTAGTACCTCCT	58	[9]
ATGL	GTCAGACGGCGAGAATGTCA	57	GGGATGAGCCCACAGTACAC	57	
PNPLA3	AGCGGTCCAGCACTTAACTC	57	GGGCCACGAAACAGTCAGTA	60	
ACOT2	ATCTGGAGTACTTTGAAGAAGCCAT	56	CCAACTCCTGGACCTTTTACCTCG	61	
CPT1A	ATGTACGCCAAGATCGACCC	57	GACATGCAGTTGGCCGTTTC	57	
HSL	AGCCTTCTGGAACATCACCGAG	60	TCGGCAGTCAGTGGCATCTCAA	60	
PPARγ	AGCCTGCGAAAGCCTTTTGGTG	68	GGCTTCACATTCAGCAAACC	65	[10]

			TGG		
CEBPA	ACGATCAGTCCATCCCAGAG	57	TTCACATTGCACAAGGCACT	57	[11,12]
PPARA	GGGATGCTGGTAGCGTATGG	60	AACTTCATGGCAAAATCAA ACTTG	53	[13]
RETN	CTAGCAAGACCCTGTGCTCC	60	CTAAATATTAGGGAGCCGG CGA	58	
PGC-1α	TTGAAAAAGCTTGACTGGCGT	54	AACCAGAGCAGCACACTCG AT	58	
CFD	TGCTACAGCTGTCGGAGAAGG	60	GCCACGTCGCAGAGAGTTC	59	
PLIN2	GATGGCAGAGAACGGTGTGAAG	62	CAGGCATAGGTATTGGCAA CTGC	64	
PLIN3	TTGCATCAGCCAGCGAATACGC	68	CACCTTAGACGACACAAGC TCC	59	
GAPDH	TGTTGCCATCAATGACCCCTT	62	CTCCACGACGTACTCAGCG	57	[14]

Supplemental Table 3: 496 LD proteins validated by quantitative proteomic analysis (Supplemental Table 3 was uploaded as "Supplementary Dataset" file)

Supplemental Table 4: Up- and downregulated LD proteins

Upregulated proteins

GN	Accession	Description	Control	Retinl	OA
PRDX1	A0A0A0MRQ5	Peroxiredoxin-1	1.0024±0.0024	1.1928±0.0754	1.2744±0.0075
WDR45	A0A0U1RQR1	WD repeat domain phosphoinositide-interacting protein 4	1.0138±0.0138	1.2244±0.0281	2.7002±0.0657
PCYT1A	C9J050	Choline-phosphate cytidylyltransferase A	1.0306±0.0306	1.0767±0.0117	1.5693±0.0507
SKP1	E5RJR5	S-phase kinase-associated protein 1	0.9348±0.0652	1.0889±0.0849	1.4325±0.0594
RUVBL1	E7ETR0	RuvB-like helicase	1.0099±0.0099	1.0349±0.025	1.1856±0.0091
HTATIP2	E9PI87	Oxidoreductase HTATIP2	0.9845±0.0155	1.2032±0.0744	1.3949±0.0645
PSMC3	E9PM69	26S protease regulatory subunit 6A	1.0186±0.0186	1.089±0.0149	1.165±0.031
HSPB1	F8WE04	Heat shock protein beta-1	0.9945±0.0055	1.1901±0.0329	1.1096±0.0197
RPS20	G3XAN0	40S ribosomal protein S20	0.965±0.035	0.9965±0.0494	1.3931±0.041
ATG2A	H7C3T2	Autophagy-related protein 2 homolog A (Fragment)	1.0572±0.0572	1.1385±0.0406	1.9855±0.065
GPX4	K7ERP4	Glutathione peroxidase (Fragment)	0.9512±0.0488	2.2308±0.0393	1.6464±0.0135
RUVBL2	M0R0Y3	RuvB-like 2	0.9765±0.0235	1.0666±0.0121	1.2915±0.0686
TPD52L2	O43399	Tumor protein D54	0.9508±0.0492	1.3839±0.221	3.774±0.0285
OPA1	O60313	Dynamin-like 120 kDa protein, mitochondrial	1.1039±0.1039	1.1325±0.1377	2.4773±0.1879
PLIN3	O60664	Perilipin-3	0.9586±0.0414	1.3121±0.3114	10.5375±0.8849
TACC1	O75410-7	Isoform 7 of Transforming acidic coiled-coil-containing protein 1	0.9883±0.0117	1.2925±0.0402	1.1768±0.0058
APOL3	O95236-3	Isoform 3 of Apolipoprotein L3	0.9965±0.0035	1.1501±0.1397	2.1518±0.045
ENO1	P06733	Alpha-enolase	0.9769±0.0231	1.3436±0.0577	1.0279±0.0377
HSP90AB1	P08238	Heat shock protein HSP 90-beta	0.9542±0.0458	1.0336±0.0335	1.0225±0.0124
RALB	P11234	Ras-related protein Ral-B	1.0396±0.0396	1.1025±0.025	1.3606±0.0267
PCNA	P12004	Proliferating cell nuclear antigen	0.9953±0.0047	1.0627±0.0249	1.2059±0.0053
CCNB1	P14635-2	Isoform 2 of G2/mitotic-specific cyclin-B1	1.0516±0.0516	1.0863±0.0005	1.377±0.0641
HMGA1	P17096-2	Isoform HMG-Y of High mobility group protein HMG-I/HMG-Y	0.9344±0.0656	1.3114±0.0019	1.0241±0.0703
LMNB1	P20700	Lamin-B1	0.9853±0.0147	1.321±0.0342	1.0589±0.0005
MSN	P26038	Moesin	0.9809±0.0191	1.2403±0.0067	0.9846±0.0181
PITPNB	P48739	Phosphatidylinositol transfer	0.9738±0.0262	1.0282±0.0027	1.3292±0.0367

		protein beta isoform			
RAB9A	P51151	Ras-related protein Rab-9A	1.0865±0.0865	1.0893±0.0502	1.7128±0.0636
RAB27A	P51159-2	Isoform Short of Ras-related protein Rab-27A	1.0675±0.0675	1.0966±0.1007	1.7726±0.0799
UBE2G2	P60604-2	Isoform 2 of Ubiquitin-conjugating enzyme E2 G2	0.9947±0.0053	1.0585±0.0297	1.1542±0.013
S100A10	P60903	Protein S100-A10	1.0324±0.0324	1.1498±0.0007	1.1925±0.0084
RAB10	P61026	Ras-related protein Rab-10	1.1145±0.1145	1.1322±0.1829	1.9028±0.0799
CALM1	P62158	Calmodulin	0.9364±0.0636	1.1533±0.0032	1.2076±0.0098
GNB2	P62879	Guanine nucleotide-binding protein G(I)/G(S)/G(T) subunit beta-2	0.996±0.004	1.0269±0.026	1.317±0.0195
RAC1	P63000	Ras-related C3 botulinum toxin substrate 1	1.0448±0.0448	1.0861±0.0741	1.4881±0.0457
BASP1	P80723	Brain acid soluble protein 1	0.9505±0.0495	1.2537±0.0417	1.2204±0.0041
RHOG	P84095	Rho-related GTP-binding protein RhoG	1.0355±0.0355	1.0894±0.0309	1.3981±0.049
LMNB2	Q03252	Lamin-B2	0.9795±0.0205	1.5179±0.146	1.5549±0.0556
AHNAK	Q09666	Neuroblast differentiation-associated protein AHNAK	0.9855±0.0145	1.1604±0.0282	1.0185±0.0006
TPRG1L	Q5T0D9	Tumor protein p63-regulated gene 1-like protein	1.0047±0.0047	1.0261±0.0215	1.5152±0.022
IKBIP	Q70UQ0-4	Isoform 4 of Inhibitor of nuclear factor kappa-B kinase-interacting protein	1.0037±0.0037	1.0705±0.0795	1.4928±0.0209
HIST2H2AB	Q8IUE6	Histone H2A type 2-B	0.962±0.038	1.5716±0.0104	1.0099±0.0343
SPG20	Q8N0X7	Spartin	0.9652±0.0348	1.2406±0.0376	1.1587±0.0251
FAM177A1	Q8N128	Protein FAM177A1	0.9888±0.0112	1.0822±0.0352	1.3005±0.0354
UFD1L	Q92890	Ubiquitin fusion degradation protein 1 homolog	0.9991±0.0009	1.2397±0.2447	1.0987±0.0417
CHMP6	Q96FZ7	Charged multivesicular body protein 6	0.9729±0.0271	1.1074±0.02	1.451±0.0238
VPS13A	Q96RL7-4	Isoform 4 of Vacuolar protein sorting-associated protein 13A	0.9866±0.0134	1.0373±0.0219	1.4773±0.0295
DESI2	Q9BSY9-2	Isoform 2 of Desumoylating isopeptidase 2	1.0837±0.0837	1.1847±0.1128	2.2161±0.0195
TRIM4	Q9C037-2	Isoform Beta of E3 ubiquitin-protein ligase TRIM4	0.9906±0.0094	1.1392±0.0711	1.6815±0.033
NDE1	Q9NXR1-2	Isoform 2 of Nuclear distribution protein nudE	0.9954±0.0046	1.3584±0.0377	1.5441±0.0286

		homolog 1			
RAB21	Q9UL25	Ras-related protein Rab-21	1.071±0.071	1.0972±0.1173	1.8702±0.0608

Down-regulated proteins

GN	Accession	Description	Control	Retinol	OA
HYOU1	A0A087X054	Hypoxia up-regulated protein 1	1.0054±0.0054	0.9778±0.0115	0.951±0.0074
NAMPT	A0A0C4DFS8	Nicotinamide phosphoribosyltransferase	1.0248±0.0248	0.9979±0.0718	0.6545±0.0011
PTPN1	B4DSN5	Tyrosine-protein phosphatase non-receptor type	1.0134±0.0134	0.9139±0.0065	0.8728±0.0125
HSD17B11	D6RCD0	Estradiol 17-beta-dehydrogenase 11	0.9396±0.0604	0.896±0.0779	0.8729±0.0726
RPLP0	F8VU65	60S acidic ribosomal protein P0 (Fragment)	0.988±0.012	0.8928±0.0156	0.8356±0.0225
ERH	G3V279	Enhancer of rudimentary homolog	1.0183±0.0183	0.996±0.011	0.8672±0.0217
PGRMC1	O00264	Membrane-associated progesterone receptor component 1	1.0259±0.0259	0.8788±0.0231	0.9352±0.0031
S100A9	P06702	Protein S100-A9	0.966±0.034	0.6922±0.0432	0.6275±0.0462
TUBB	P07437	Tubulin beta chain	0.9736±0.0264	0.9118±0.005	0.7±0.0003
EEF2	P13639	Elongation factor 2	0.9982±0.0018	0.8922±0.0216	0.9675±0.0076
PKM	P14618	Pyruvate kinase PKM	0.9828±0.0172	0.9631±0.0164	0.8902±0.003
ATP2A2	P16615-2	Isoform 2 of Sarcoplasmic/endoplasmic reticulum calcium ATPase 2	1.0708±0.0708	0.7096±0.0285	1.0224±0.0336
CANX	P27824	Calnexin	1.0425±0.0425	0.6904±0.0094	0.9446±0.0434
MYH9	P35579	Myosin-9	0.9998±0.0002	0.953±0.0077	0.8635±0.0375
DDOST	P39656-2	Isoform 2 of Dolichyl-diphosphooligosaccharide--protein glycosyltransferase 48 kDa subunit	0.981±0.019	0.7553±0.0104	0.8776±0.0095
RPL5	P46777	60S ribosomal protein L5	0.9739±0.0261	0.9438±0.0033	0.8616±0.0013
BCAP31	P51572	B-cell receptor-associated protein 31	1.0673±0.0673	0.692±0.0387	0.9681±0.0173
EEF1A1	P68104	Elongation factor 1-alpha 1	0.9897±0.0103	0.9305±0.0312	0.5858±0.0195
TUBA1B	P68363	Tubulin alpha-1B chain	1.0035±0.0035	0.8295±0.0461	0.5069±0.0328
MFGE8	Q08431-2	Lactadherin	0.8±0.2	0.4±0.1	0.15±0.05
FLNC	Q14315-2	Isoform 2 of Filamin-C	0.9896±0.0104	0.9733±0.0068	0.9137±0.0012
PCBP2	Q15366-6	Isoform 6 of Poly(rC)-binding protein 2	1.0239±0.0239	0.7453±0.0355	0.7216±0.0512
ATL3	Q6DD88	Atlastin-3	1.0597±0.0597	0.7506±0.0259	1.0173±0.0025
TUBA1A	Q71U36-2	Isoform 2 of Tubulin alpha-1A chain	0.9867±0.0133	0.9604±0.0209	0.7836±0.0178
NPLOC4	Q8TAT6	Nuclear protein localization protein 4 homolog	0.9784±0.0216	0.9538±0.0054	0.6037±0.0035
KIF16B	Q96L93-6	Isoform 5 of Kinesin-like protein	1.0329±0.0329	0.7482±0.0282	0.7717±0.0037

		KIF16B			
TUBB6	Q9BUF5	Tubulin beta-6 chain	0.9844 ±0.0156	0.9598 ±0.0059	0.7644 ±0.0374
RAB18	Q9NP72	Ras-related protein Rab-18	1 ±0	0.7727 ±0.1364	0.2273 ±0.0455

1 Chen, L. *et al.* Snail Driving Alternative Splicing of CD44 by ESRP1 Enhances Invasion and Migration in Epithelial Ovarian Cancer. *Cellular physiology and biochemistry : international journal of experimental cellular physiology, biochemistry, and pharmacology* **43**, 2489-2504, doi:10.1159/000484458 (2017).

2 Barth, K. A., Waterfield, J. D. & Brunette, D. M. The effect of surface roughness on RAW 264.7 macrophage phenotype. *Journal of biomedical materials research. Part A* **101**, 2679-2688, doi:10.1002/jbm.a.34562 (2013).

3 Tarzemany, R., Jiang, G., Jiang, J. X., Larjava, H. & Hakkinen, L. Connexin 43 Hemichannels Regulate the Expression of Wound Healing-Associated Genes in Human Gingival Fibroblasts. *Sci Rep* **7**, 14157, doi:10.1038/s41598-017-12672-1 (2017).

4 Intriago, B. & Fernanda, M. Regulation of gingival fibroblast gene expression by leukocyte and platelet rich fibrin. *University of British Columbia* (2017).

5 Khodyuchenko, T. *et al.* Congenital heart defects are rarely caused by mutations in cardiac and smooth muscle actin genes. *BioMed research international* **2015**, 127807, doi:10.1155/2015/127807 (2015).

6 Li, C. P., Li, H. J., Nie, J., Chen, X. & Zhou, X. Mutation of miR-21 targets endogenous lipoprotein receptor-related protein 6 and nonalcoholic fatty liver disease. *Am J Transl Res* **9**, 715-721 (2017).

7 Go, G. W. *et al.* t10,c12 conjugated linoleic acid upregulates hepatic de novo lipogenesis and triglyceride synthesis via mTOR pathway activation. *Journal of microbiology and biotechnology* **23**, 1569-1576 (2013).

8 Go, G. W. *et al.* The combined hyperlipidemia caused by impaired Wnt-LRP6 signaling is reversed by Wnt3a rescue. *Cell metabolism* **19**, 209-220, doi:10.1016/j.cmet.2013.11.023 (2014).

9 Shih, C. & Huang, J.-Y. Treating hepatitis virus infection by modulating micrornas mir-130a, mir-130b, mir-204, or mir-1236. *Academia Sinica* (2016).

10 Ouyang, H. *et al.* WNT7A and PAX6 define corneal epithelium homeostasis and pathogenesis. *Nature* **511**, 358-361, doi:10.1038/nature13465 (2014).

11 Scaffidi, P. & Misteli, T. Lamin A-dependent misregulation of adult stem cells associated with accelerated ageing. *Nat Cell Biol* **10**, 452-459, doi:10.1038/ncb1708 (2008).

12 Yin, S., Luan, J., Fu, S., Wang, Q. & Zhuang, Q. Does Water-Jet Force Make a Difference in Fat Grafting? In Vitro and In Vivo Evidence of Improved Lipoaspirate Viability and Fat Graft Survival. *Plastic and Reconstructive Surgery* **135**, 127-138, doi:10.1097/prs.0000000000000780 (2015).

13 Hiramitsu, M. *et al.* Eriocitrin ameliorates diet-induced hepatic steatosis with activation of mitochondrial biogenesis. *Sci Rep* **4**, 3708, doi:10.1038/srep03708 (2014).

14 Tangchitphisut, P. *et al.* Infrapatellar Fat Pad: An Alternative Source of Adipose-Derived Mesenchymal Stem Cells. *Arthritis* **2016**, 4019873, doi:10.1155/2016/4019873 (2016).
